# Supplementary material for: Quantitative three-dimensional local order analysis of nanomaterials through electron diffraction
Source: Nat Commun. 2023 Oct 16;14:6512. doi: 10.1038/s41467-023-41934-y (PMC10579245; doi:10.1038/s41467-023-41934-y)
Supplement: Supplementary file 1 — Supplementary Info [file 41467_2023_41934_MOESM1_ESM.pdf]

Supplementary Information

Quantitative three-dimensional local order analysis of nanomaterials through electron diffraction

Ella M. Schmidt, *et al.*

Contents

Supplementary Methods 1: Parameters for data treatment **S3**

X-ray and neutron diffraction S3

Electron diffraction S3

Supplementary Note 1: Sample preparation and 3D electron diffraction measurement **S5**

Supplementary Note 2: Reciprocal space coverage **S7**

Supplementary Note 3: Two dimensional sections of the shortest vectors **S9**

Supplementary Note 4: Three dimensional fits **S11**

$\left(\frac{1}{2}, 0, 0\right)$ interatomic vector S11

$\left(\frac{1}{4}, \frac{1}{4}, \frac{1}{4}\right)$ interatomic vector S13

$\left(\frac{1}{2}, \frac{1}{2}, 0\right)$ interatomic vector S13

Supplementary Note 5: Model **S15**

Supplementary Discussion 1: Maximum observable correlation lengths **S18**

Supplementary Discussion 2: Relative ratio of the form factors **S20**

Supplementary Discussion 3: Discussion of the deviations in determined shift magnitudes **S21**

Scattering lengths S21

Determination of shift magnitudes from maxima and minima in the 3D-ΔPDF S22

Supplementary References **S25**

Supplementary Methods 1: Parameters for data treatment

X-ray and neutron diffraction

The data treatment for x-ray and neutron diffraction experiments was identical. Reciprocal space was reconstructed on a grid of $501 \times 501 \times 501$ voxels with $-10 \leq h, k, l \leq 10$. For the Karen[S6] algorithm a median window width of 5 voxels was chosen. The Bragg peaks that fulfil the F-centring diffraction conditions were punched with a sphere with a punch radius of 8 voxels. To interpolate the missing intensity the SciPy[S5] interpolation of grid-data was used with a mutiquadratic function (Rbf with parameters smooth=2.5, epsilon=2.5). For the Gaussian falloff to reduce finite size ripples in the Fourier transform for each voxel the distance d in voxels to the centre of reciprocal space was calculated and its intensity multiplied with $\exp(-7.5 \cdot 10^{-5} \cdot d^2)$.

Electron diffraction

Reciprocal space was reconstructed on a grid of $201 \times 201 \times 201$ voxels with $-10 \leq h, k, l \leq 10$. For the Karen[S6] algorithm a median window width of 5 voxels was chosen. The Bragg peaks that fulfil the F-centring diffraction conditions were punched with a sphere with a punch radius of 5 voxels. On an absolute scale this corresponds to a much larger sphere than in the case for x-ray and neutron diffraction. To interpolate the missing intensity the SciPy[S5] interpolation of grid-data was used with a mutiquadratic function (Rbf with parameters smooth=2.5, epsilon=2.5). For the Gaussian falloff to reduce finite size ripples in the Fourier transform for each voxel the distance d in voxels to the centre of reciprocal space was calculated and its intensity multiplied with $\exp(-1.25 \cdot 10^{-4} \cdot d^2)$. This is the same relative dampening as applied to the x-ray and neutron data.

The effect of different punch radii is shown in Supplementary Figure 1. With a punch radius of only 3 voxels in the case of electron diffraction not all of the Bragg peak intensities are removed, which results in strong maxima in the 3D-ΔPDF at average structure interatomic vectors. For a punch radius of 5 voxels this effect is removed, while for even larger punch radii no visible difference is observed. The punch radius was chosen here as the minimum punch radius for which in real space no clear positive density in the 3D-ΔPDF is observed at the majority of average structure interatomic vectors.

Supplementary Figure 1: The effect of different punch radii on the data processing. Left column $r_{punch} = 3$ voxels, middle column $r_{punch} = 5$ voxels, right column $r_{punch} = 7$ voxels. Top $hk0$-layer after punching, middle $hk0$-layer after interpolation and application of Gaussian falloff, bottom 3D-ΔPDFs in the $ab0$-layer.

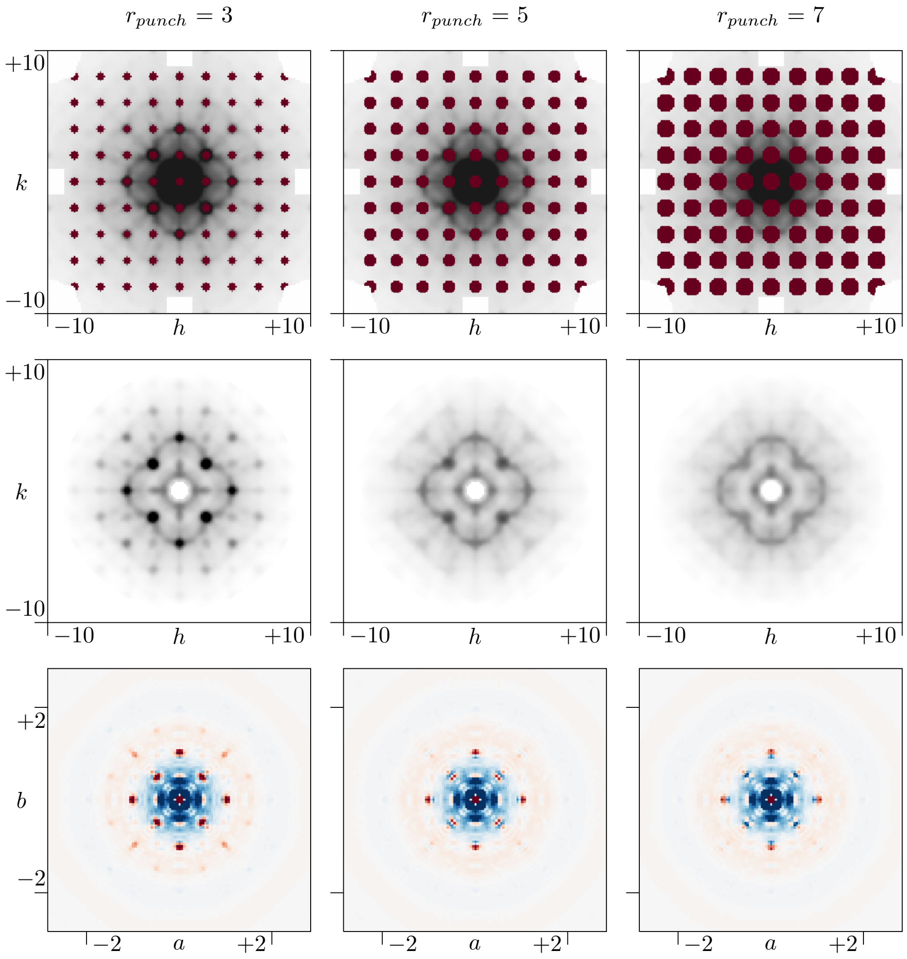

The electron diffraction experiment did not use a beam stop. Therefore the central region of reciprocal space also includes a halo of the primary electron beam on the detector. To subtract the contributions of the primary beam the punched and filled data was radially integrated and approximated with a sum of two Gaussian functions. The full width at half maximum (FWHM) of these Gaussians is much wider than for typical diffuse scattering features. This background was subsequently subtracted from the diffraction data before Fourier transforming.

Supplementary Note 1: Sample preparation and 3D electron diffraction measurement

Initial test measurements used a crushed sample. However, a suitable crystal could not be found as crystallites on the grid were too thick so that Kikuchi lines were present in the diffraction patterns (Supplementary Figure 2). Therefore, a sample with an optimised thickness of about 50 nm was prepared by ion milling as described in the Methods part of the main text. A side view of the ion milled samples is shown in Supplementary Figure 3.

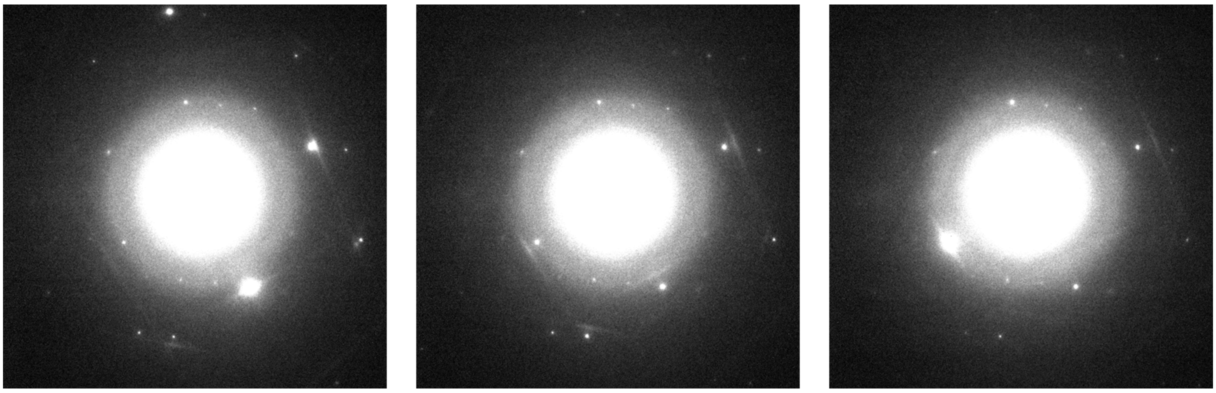

Supplementary Figure 2: Diffraction patterns of a crystallite prepared by simply crushing the sample. The goniometer orientation angles are -50.0° (left), -49.75° (middle), and -49.5° (right). Kikuchi lines are clearly visible, and these are much stronger than any diffuse scattering (not visible here).

Supplementary Figure 4 shows the top view of the ion milled sample prepared for the electron diffraction experiments. Residual Ga ions from ion milling could potentially be incorporated on the sample surface and hence reduce longer range coherence within the sample.

To further confirm the estimated sample thickness, another data set was recorded with parameters optimised for average structure determination (210 ms exposure per frame with 0.5° goniometer rotation from -50° to +50°). The dynamical refinement (Robs = 11.2 % for 580 observed reflections) was mainly used to estimate the thickness, which refined to a value of 49.2(6) nm. This is in good agreement with the target thickness from the sample preparation.

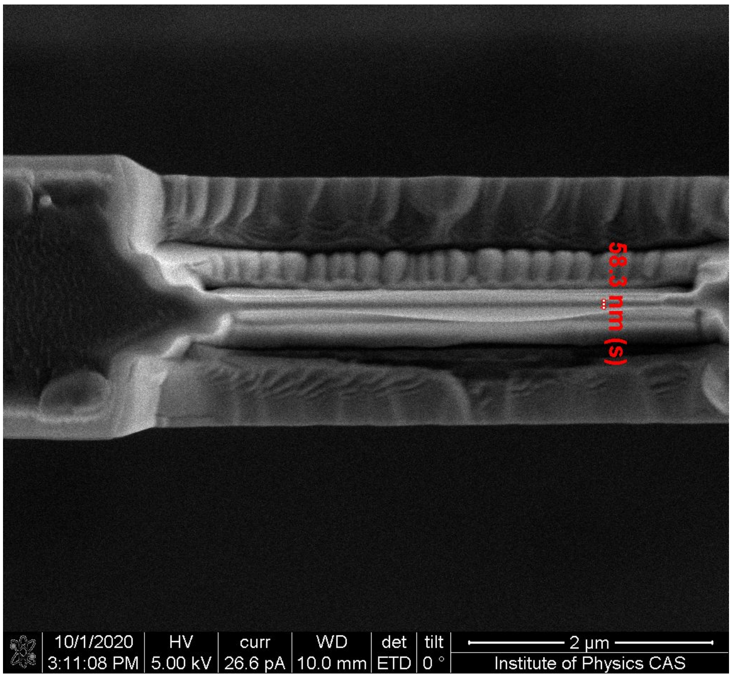

Supplementary Figure 3: SEM image of the ion milled sample prepared for electron diffraction experiments.

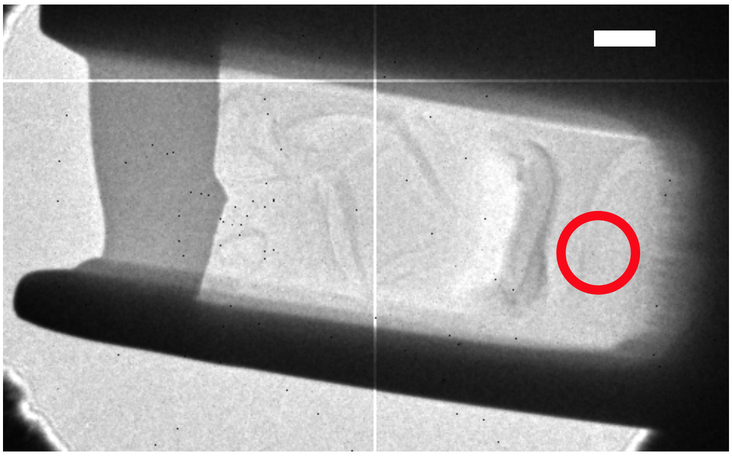

Supplementary Figure 4: Transmission electron microscopy image of the lamella. Scale bar at top right is 1 μm. The red circle marks the area illuminated during data collection. Crystal tracking was used to make the beam follow the movement of the sample stage during the goniometer rotation.

Supplementary Note 2: Reciprocal space coverage

Supplementary Figure 5 shows the reciprocal space coverage before and after symmetry averaging for the three performed diffraction experiments. The unprocessed $hk0$, $h0l$ and $0kl$ layers after reconstruction are shown in Supplementary Figure 6. In the electron diffraction experiment approximately 28 % of reciprocal space were measured. Due to the limited goniometer tilt range the missing wedge in the data is clearly observed. The neutron diffraction experiment was conducted using a 180 deg ϕ-scan, which yields a reciprocal space coverage of approximately 50 %. For x-ray diffraction several ω-scans were conducted, which yield 100 % reciprocal space coverage in the considered resolution range. The high $m\bar{3}m$ Laue symmetry allows to fill any remaining gaps by symmetry equivalent data.

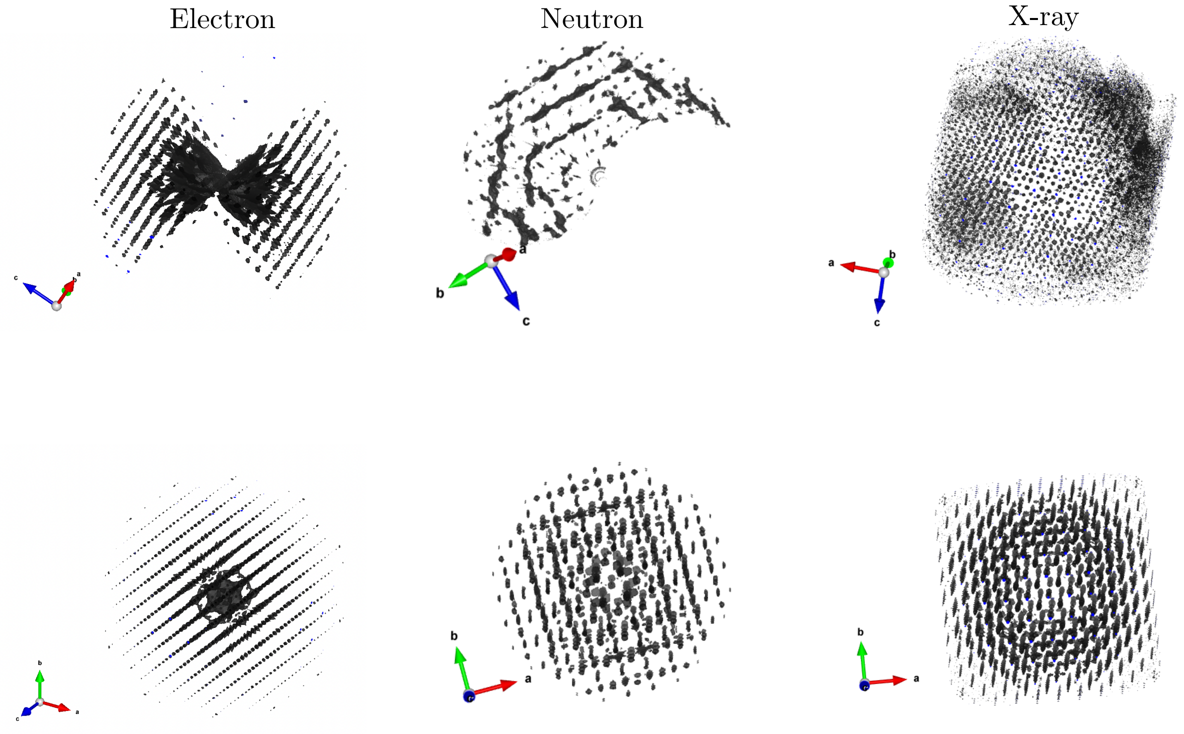

Supplementary Figure 5: Three dimensional renderings of the reconstructed diffraction space for the electron, neutron and x-ray diffraction experiment. Top before and bottom after symmetry averaging for $m\bar{3}m$ Laue symmetry. Note that the direction labels correspond to the reciprocal space axis $\vec{a}^*$ in red, $\vec{b}^*$ in green and $\vec{c}^*$ in blue.

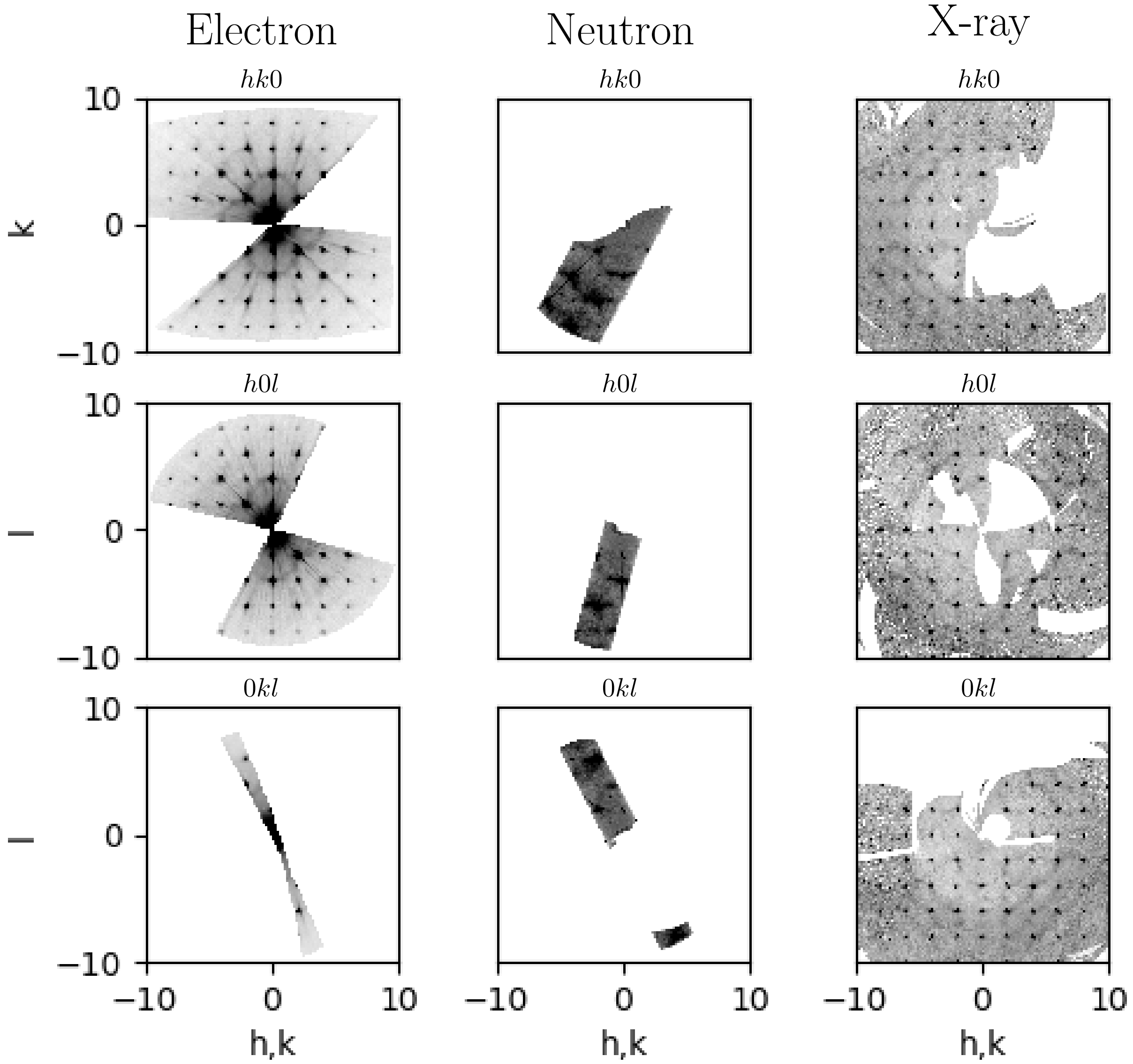

Supplementary Figure 6: Unprocessed reconstructions. Top $hk0$, middle $h0l$ and bottom $0kl$ from electron (left), neutron (middle) and x-ray (right) diffraction experiments.

Supplementary Note 3: Two dimensional sections of the shortest vectors

Two dimensional sections of the shortest interatomic vectors are shown in Supplementary Figures 7-10.

Supplementary Figure 7: 3D-ΔPDFs in the $ab0$-layer. 3D-ΔPDFsobtained from x-ray (left), neutron (middle) and electron (right) diffraction experiments. Two-dimensional plot in the $ab0$-layer. Dashed lines indicate the average interatomic vector at $\left(\frac{1}{2}, 0, 0\right)$. Positive intensities in red, negative intensities in blue.

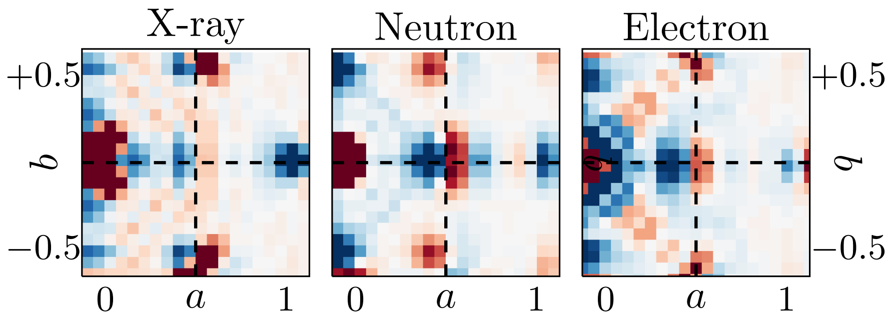

Supplementary Figure 8: 3D-ΔPDFs in the $ab0.25$-layer. 3D-ΔPDFs obtained from x-ray (left), neutron (middle) and electron (right) diffraction experiments. Two-dimensional plot in the $ab0.25$-layer. Dashed lines indicate the average interatomic vector at $\left(\frac{1}{4}, \frac{1}{4}, \frac{1}{4}\right)$. Positive intensities in red, negative intensities in blue.

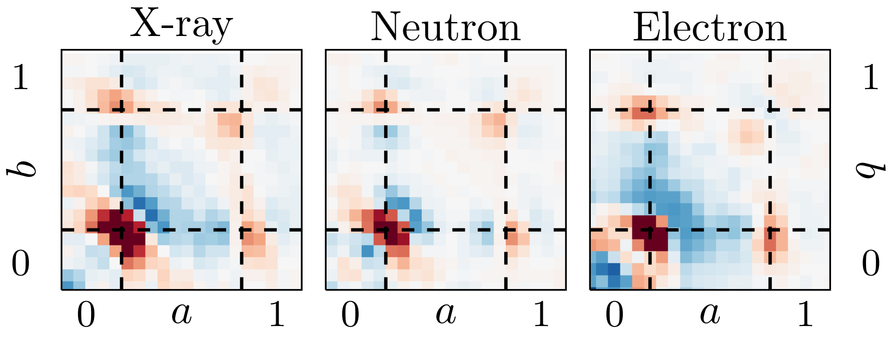

Supplementary Figure 9: 3D-ΔPDFs in the $ab0$-layer. 3D-ΔPDFs obtained from x-ray (left), neutron (middle) and electron (right) diffraction experiments. Two-dimensional plot in the $ab0$-layer. Dashed lines indicate the average interatomic vector at $\left(\frac{1}{2}, \frac{1}{2}, 0\right)$. Positive intensities in red, negative intensities in blue.

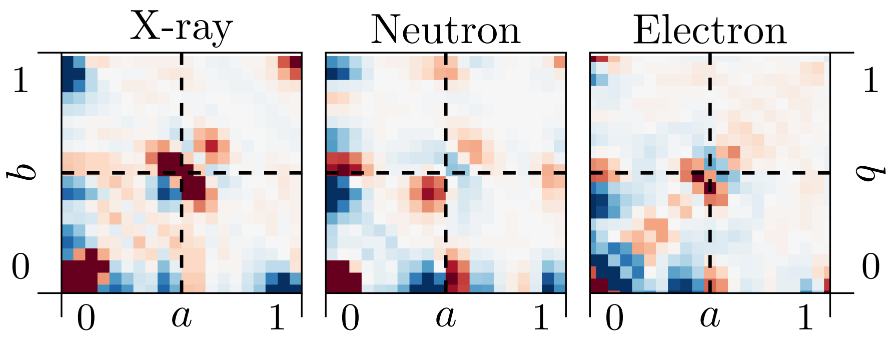

Supplementary Figure 10: 3D-ΔPDFs in the $ab0.5$-layer. 3D-ΔPDFs obtained from x-ray (left), neutron (middle) and electron (right) diffraction experiments. Two-dimensional plot in the $ab0.5$-layer. Dashed lines indicate the average interatomic vector at $\left(\frac{1}{2}, \frac{1}{2}, \frac{1}{2}\right)$. Positive intensities in red, negative intensities in blue.

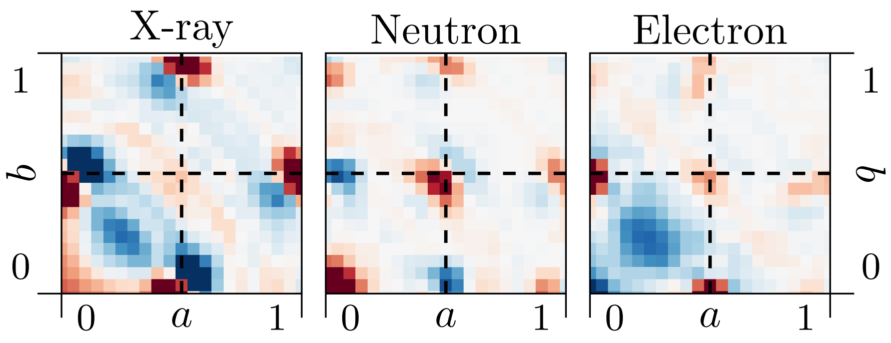

Supplementary Note 4: Three dimensional fits

The position of several maxima and minima in the 3D-Δ PDFs were refined by approximating the intensity distribution as three-dimensional Gaussian distributions. For this purpose the Voronoi cell around each of the shortest interatomic vectors was extracted from the data. Positive and negative intensities were treated separately. For all interatomic vectors a function of the form

$$I(\vec{x}) = I_0 \cdot \exp\left(-\frac{1}{2}(\vec{x}-\vec{x}_0)^T \underline{\underline{M}}(\vec{x}-\vec{x}_0)\right) \tag{1}$$

was fitted to the intensity distribution.

To address the effect of the chosen reconstruction grid in reciprocal space we also processed the x-ray and neutron data on the 201 × 201 × 201 voxel grid as for the electron data. The corresponding fit values are reported accordingly and are within the standard deviations of the higher reciprocal space resolution data. Note that the data processing did not keep absolute units and therefore the fitted intensity values are not comparable between the different reconstructions.

$\left(\frac{1}{2}, 0, 0\right)$ interatomic vector

As the observed intensity distribution around the $\left(\frac{1}{2}, 0, 0\right)$ interatomic vector is rotationally symmetric around the a-axis the number of free parameters in Equation 1 was reduced to four: $\vec{x}_0$ was restricted to $\left(\frac{1}{2}+\Delta, 0, 0\right)$ and $\underline{\underline{M}}$ was restricted to

$$\underline{\underline{M}} = \begin{pmatrix} m_{11} & 0 & 0 \\ 0 & m_{22} & 0 \\ 0 & 0 & m_{22} \end{pmatrix}.$$

The resulting parameters are listed in Supplementary Table 1.

Supplementary Table 1: Fit fo the $\left(\frac{1}{2},0,0\right)$ interatomic vector. Refined parameters for the Gaussian distribution the observed maxima and minima away from the average $\left(\frac{1}{2},0,0\right)$ interatomic vector.

	I_0 [arb. u.]	Δ^+ [r.l.u.]	m_{11} [(r.l.u)$^{-2}$]	m_{22} [(r.l.u)$^{-2}$]
		Maximum		
e^-	3.10(109)	$2.19(16) \cdot 10^{-2}$	$2.29(114) \cdot 10^{3}$	$1.66(4) \cdot 10^{2}$
γ (501 voxels)	0.481(29)	$2.28(17) \cdot 10^{-2}$	$0.71(8) \cdot 10^{3}$	$0.63(7) \cdot 10^{2}$
γ (201 voxels)	3.30(19)	$2.34(16) \cdot 10^{-2}$	$0.73(8) \cdot 10^{3}$	$0.64(6) \cdot 10^{2}$
n (501 voxels)	5.36(13)	$1.71(5) \cdot 10^{-2}$	$1.09(5) \cdot 10^{3}$	$1.03(3) \cdot 10^{2}$
n (201 voxels)	3.42(8)	$1.71(5) \cdot 10^{-2}$	$1.09(5) \cdot 10^{3}$	$1.03(3) \cdot 10^{2}$
		Minimum		
e^-	-3.36(7)	$-1.17(1) \cdot 10^{-1}$	$3.19(19) \cdot 10^{2}$	$1.39(4) \cdot 10^{2}$
γ (501 voxels)	-1.31(3)	$-0.92(1) \cdot 10^{-1}$	$12.06(53) \cdot 10^{2}$	$1.38(4) \cdot 10^{2}$
γ (201 voxels)	-8.43(20)	$-0.92(9) \cdot 10^{-1}$	$12.55(55) \cdot 10^{2}$	$1.38(5) \cdot 10^{2}$
n (501 voxels)	-7.83(25)	$-1.02(1) \cdot 10^{-1}$	$6.22(3) \cdot 10^{2}$	$3.11(13) \cdot 10^{2}$
n (201 voxels)	-5.00(16)	$-1.02(1) \cdot 10^{-1}$	$6.22(3) \cdot 10^{2}$	$3.11(13) \cdot 10^{2}$

$\left(\frac{1}{4}, \frac{1}{4}, \frac{1}{4}\right)$ interatomic vector

For the purpose of the fit the intensity distribution was approximated as rotationally symmetric around the [111]-direction. The number of free parameters was reduced to four: $\vec{x}_0$ was restricted to $\left(\frac{1}{4} + \Delta, \frac{1}{4} + \Delta, \frac{1}{4} + \Delta\right)$ and $\underline{\underline{M}}$ was restricted to

$$\underline{\underline{M}} = \begin{pmatrix} m_{11} & m_{12} & m_{12} \\ m_{12} & m_{11} & m_{12} \\ m_{12} & m_{12} & m_{11} \end{pmatrix}.$$

The resulting parameters are listed in Supplementary Table 2.

Supplementary Table 2: Fit fo the $\left(\frac{1}{4}, \frac{1}{4}, \frac{1}{4}\right)$ interatomic vector. Refined parameters for the Gaussian distribution the observed maxima and minima away from the average $\left(\frac{1}{4}, \frac{1}{4}, \frac{1}{4}\right)$ interatomic vector.

	I_0 [arb. u.]	Δ^+ [r.l.u.]	m_{11} [(r.l.u)$^{-2}$]	m_{12} [(r.l.u)$^{-2}$]
		Maximum		
e^-	10.3(3)	$-1.29(5) \cdot 10^{-2}$	$4.95(17) \cdot 10^2$	$2.35(16) \cdot 10^2$
γ (501 voxels)	2.85(8)	$-1.59(6) \cdot 10^{-2}$	$3.18(12) \cdot 10^2$	$1.54(11) \cdot 10^2$
γ (201 voxels)	19.1(5)	$-1.55(6) \cdot 10^{-2}$	$3.20(11) \cdot 10^2$	$1.51(11) \cdot 10^2$
n (501 voxels)	7.31(24)	$-9.75(52) \cdot 10^{-2}$	$5.13(22) \cdot 10^2$	$2.72(21) \cdot 10^2$
n (201 voxels)	4.68(15)	$-9.75(52) \cdot 10^{-2}$	$5.13(22) \cdot 10^2$	$2.72(21) \cdot 10^2$
		Minimum		
e^-	-2.71(13)	$5.93(12) \cdot 10^{-2}$	$1.85(13) \cdot 10^2$	$1.13(12) \cdot 10^2$
γ (501 voxels)	-1.44(6)	$6.45(9) \cdot 10^{-2}$	$2.45(13) \cdot 10^2$	$1.32(12) \cdot 10^2$
γ (201 voxels)	-9.17(37)	$6.49(9) \cdot 10^{-2}$	$2.37(13) \cdot 10^2$	$1.26(12) \cdot 10^2$
n (501 voxels)	-2.88(28)	$4.98(14) \cdot 10^{-2}$	$4.76(66) \cdot 10^2$	$3.36(65) \cdot 10^2$
n (201 voxels)	-1.84(17)	$4.98(14) \cdot 10^{-2}$	$4.76(66) \cdot 10^2$	$3.36(64) \cdot 10^2$

$\left(\frac{1}{2}, \frac{1}{2}, 0\right)$ interatomic vector

In the neutron diffraction experiment the signatures of O-O correlations and those of metal-metal correlations are observable on similar absolute scales as the scattering lengths of oxygen

and the metals are comparable. For x-ray and electron diffraction the metals dominate the 3D-ΔPDF map. We interpret the clear maximum at $\left(\frac{1}{2}+\Delta, \frac{1}{2}+\Delta, 0\right)$ as the signature of two metal ions that are both relaxed away along their respective $\langle 111\rangle$ directions from a bridging vacancy. We refine the position of this maximum using a Gaussian distribution as described in Equation 1. To avoid the influence of the second larger maximum on the fit, the data in the extracted voronoi cell is constrained to $a, b \geq 0.5$. In the neutron diffraction experiment, the signature shows a different shape, which is a clear indication that the oxygen ions also contribute in this case, hence a resulting fit position would not be comparable.

The number of free parameters was reduced to five: $\vec{x}_0$ was restricted to $\left(\frac{1}{2}+\Delta, \frac{1}{2}+\Delta, 0\right)$ and $\underline{\underline{M}}$ was restricted to

$$\underline{\underline{M}} = \begin{pmatrix} m_{11} & m_{12} & 0 \\ m_{12} & m_{11} & 0 \\ 0 & 0 & m_{33} \end{pmatrix}.$$

The resulting parameters are listed in Supplementary Table 3.

Supplementary Table 3: Fit fo the $\left(\frac{1}{2}, \frac{1}{2}, 0\right)$ interatomic vector. Refined parameters for the Gaussian distribution the observed maxima away from the average $\left(\frac{1}{2}, \frac{1}{2}, 0\right)$ interatomic vector.

	I_0 [arb. u.]	Δ^+ [r.l.u.]	m_{11} [(r.l.u)$^{-2}$]	m_{33} [(r.l.u)$^{-2}$]	m_{12} [(r.l.u)$^{-2}$]]
e^-	2.52(5)	$8.74(4) \cdot 10^{-2}$	$7.55(20) \cdot 10^2$	$6.69(20) \cdot 10^2$	$2.74(20) \cdot 10^2$
γ (501 voxels)	1.24(3)	$10.43(4) \cdot 10^{-2}$	$6.87(20) \cdot 10^2$	$7.03(24) \cdot 10^2$	$2.77(21) \cdot 10^2$
γ (201 voxels)	7.98(71)	$10.39(5) \cdot 10^{-2}$	$6.92(20) \cdot 10^2$	$7.13(24) \cdot 10^2$	$2.72(21) \cdot 10^2$

Supplementary Note 5: Model

We use the model that we established in our previous work[S3] to show that this also fits the electron diffraction results. We used Discus[S1] to calculate the scattering in the kinematic approximation. The resulting reciprocal $hk0$-layer, hhl-layer and real space $ab0$- and aac-sections are shown in Supplementary Figures 11 and 12. Two-dimensional sections of the calculated and experimentally obtained 3D-ΔPDF around the shortest interatomic vectors are compared in Supplementary Figure 13. Supplementary Figure 14 compares the three-dimensional renderings of the model calculated for all three radiation types to the respective data.

Supplementary Figure 11: Comparison of data and model in reciprocal space. Observed diffuse scattering compared to the calculated diffraction pattern of the model for electron diffraction applying the kinematic approximation. (left) $hk0$-layer,(right) hhl-layer.

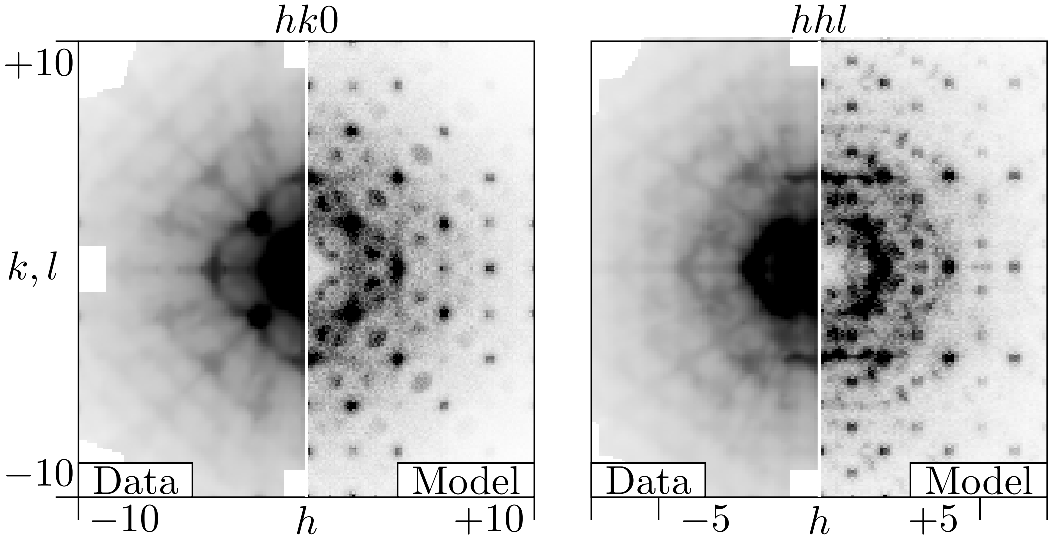

Supplementary Figure 12: Comparison of data and model in real space. 3D-ΔPDF maps from the electron diffraction experiment compared to the model in kinematic approximation. (left) $ab0$-layer,(right) aac-layer.

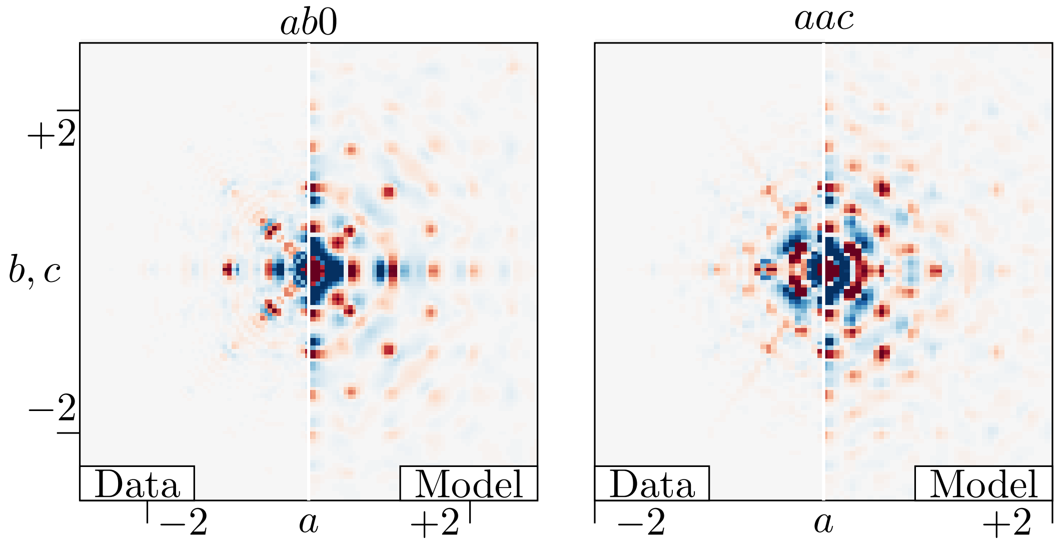

Supplementary Figure 13: Comparison of data and model in real space. 3D-ΔPDFs obtained from a simplified model (M) compared to the experimentally obtained 3D-ΔPDFs (E) for electron diffraction. Three dimensional renderings are compared in Figure 4 of the main text. (a) Section around $(\frac{1}{2}, 0, 0)$ in the $ab0$-layer, model top, experiment bottom. (b) Section around $(\frac{1}{4}, \frac{1}{4}, \frac{1}{4},)$ in the $ab\frac{1}{4}$-layer, model top left, experiment bottom right. (c) Two-dimensional section around $(\frac{1}{2}, \frac{1}{2}, 0)$ in the $ab0$-layer model top left, experiment bottom right. (d) Two-dimensional section around $(\frac{1}{2}, \frac{1}{2}, \frac{1}{2})$ in the $ab\frac{1}{2}$-layer, model top left, experiment bottom right

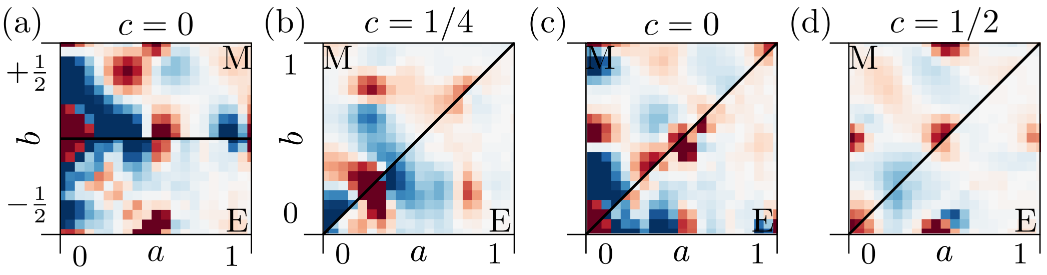

Supplementary Figure 14: Three-dimensional renderings of the model. Three-dimensional renderings of the 3D-ΔPDFs calculated from the simplified model described in[S3] compared to the experimental data for all three radiation types used.

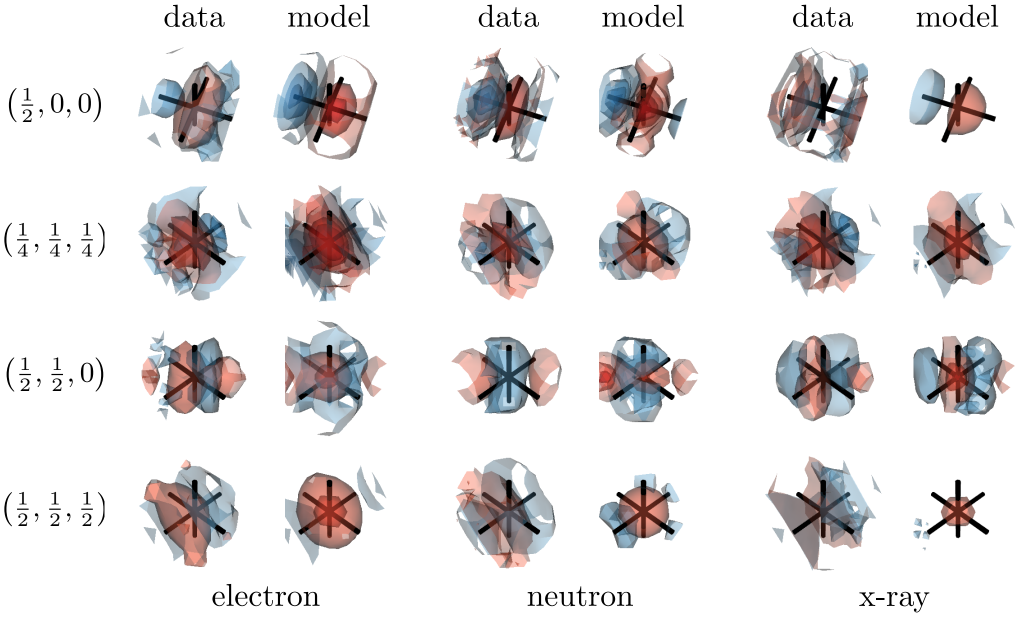

Supplementary Discussion 1: Maximum observable correlation lengths

As described in the main text the observed densities in the electron diffraction 3D-ΔPDF fall of much faster then the corresponding neutron and x-ray 3D-ΔPDFs. We attribute the main cause of this to the experimentally observed broadening in the electron diffraction experiment. To quantify this, we estimate the width of the (022) Bragg reflection in the reconstructions after symmetry averaging and compare this width to the width of a sharp diffuse feature - namely a cut through the diffuse arc at about (1.9 2.6 0). Supplementary Figure 15(a) show the normalized line cuts along $h22$ through the (022) Bragg reflections and fitted Gaussian functions to evaluate the $\mathrm{FWHM}_{\mathrm{Bragg}}$. The estimated $\mathrm{FWHM}_{\mathrm{Bragg}}$ and corresponding correlation lengths are listed in Supplementary Table 4. In an ideal experiment and an ideal reconstruction the Bragg reflections would be exactly one voxel. This is not the case and hence we use the $\mathrm{FWHM}_{\mathrm{Bragg}}$ determined here as an approximation for an instrumental resolution function.

Supplementary Table 4: Maximum observable and structural correlation lengths. Estimated maximum observable correlation lengths from the width of the (022) Bragg reflection and the diffuse arc at ≈ (1.9 2.6 0) and deduced structural correlation length ξ_{stru} in relative lattice units..

	$\mathrm{FWHM}_{\mathrm{Bragg}}$ [(r.l.u.)$^{-}$1]	ξ_{Bragg} [r.l.u]	$\mathrm{FWHM}_{\mathrm{Diff.}}$ [(r.l.u.)$^{-}$1]	$\xi_{\mathrm{Diff.}}$ [r.l.u]	ξ_{stru} [r.l.u]
e^-	0.24 ± 0.10	4.16 ± 1.75	0.91 ± 0.13	1.09 ± 0.16	1.14 ± 0.18
n	0.045 ± 0.022	22.3 ± 10.8	0.35 ± 0.13	2.81 ± 1.01	2.84 ± 1.04
γ	0.071 ± 0.037	14.0 ± 7.4	0.53 ± 0.10	1.89 ± 0.37	1.91 ± 0.38

Generally, the diffuse scattering is broader than the Bragg reflections and the width of the diffuse scattering is an indication for the extend of local order. To estimate the extend of the local order, we estimate the width of a relatively sharp diffuse feature - the diffuse arc at ≈ (1.9 2.6 0). Normalized line cuts through this arc are shown in Supplementary Figure 15(b). The intensity is fitted with a Gaussian function and a linear background. The refined $\mathrm{FWHM}_{\mathrm{Diff.}}$ of the diffuse scattering is listed in Supplementary Table 4.

To disentangle the influence of the structure related broadening of the diffuse scattering (ξ_{stru}) and the instrumental setup related broadening, we assume that the measured diffuse

Supplementary Figure 15: Determination of the width of reciprocal space features. (a) Normalized line cut of the symmetry averaged reconstruction along h 2 2 for electron (green), x-ray (purple) and neutron (blue) diffraction. Dashed lines indicate the fit of a Gaussian with fixed maximum intensity and fixed position at $h = 0$. (b) Normalized line cut of the final diffuse scattering reconstruction along h 2.6 0 for electron (green), x-ray (purple) and neutron (blue) diffraction. Dashed lines indicate the fit of a Gaussian with a linear background function.

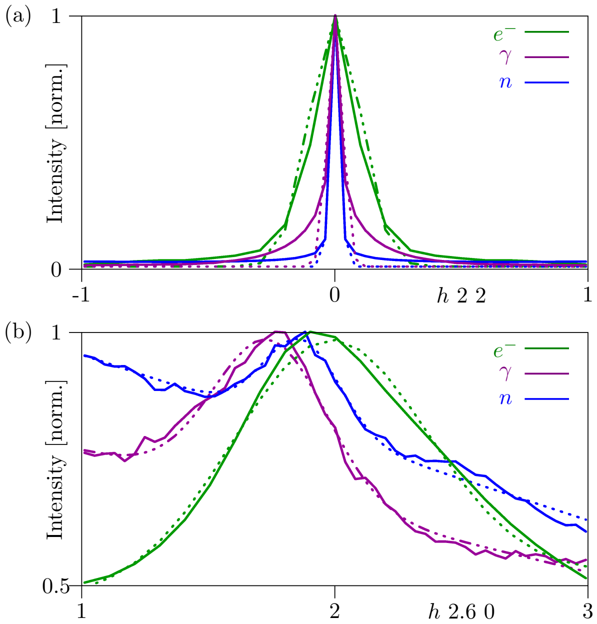

scattering width is the convolution of the experimental setup related broadening as described by our Bragg data analysis and the actual structural diffuse scattering. The derived structural correlation length is listed in Supplementary Table 4.

From this we conclude that the estimated structural correlation length for the electron diffraction experiment is reduced compared to the x-ray and neutron correlation lengths but only within two standard deviations. Hence, we attribute the main cause for the faster falloff of the observed densities for electron diffraction 3D-ΔPDF as compared to the corresponding neutron and x-ray 3D-ΔPDFs to the experimental setup related broadening of the observed diffraction patterns, while structural alteration do to the harsh sample preparation only plays a minor role.

Supplementary Discussion 2: Relative ratio of the form factors

In Supplementary Figure 16 the relative scattering power of O vs. Zr is visualised for the three different radiation types in the resolution range used in the experiments. Higher f(O)/f(Zr) or b(O)/b(Zr) indicates a contrast that is more favourable to detect lighter elements. Form factors were calculated using Table 4.3.2.2 in the International Tables for Crystallography vol C[S2].

Supplementary Figure 16: Comparison of ratios of form factors and neutron scattering lengths. Ratio of atomic form factors and neutron scattering lengths for O vs. Zr for the three radiation types used in the experiment.

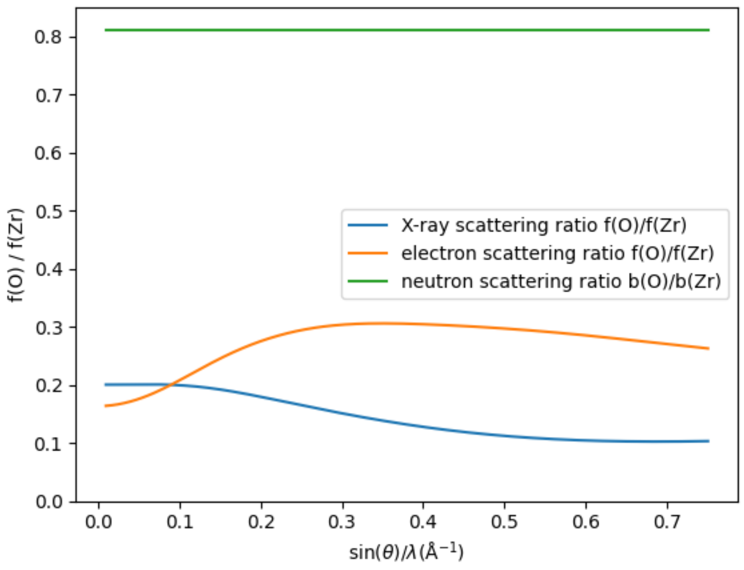

Supplementary Discussion 3: Discussion of the deviations in determined shift magnitudes

Scattering lengths

Figure 4 of the main text shows the shift of the experimentally observed minima and maxima in the 3D-ΔPDF densities. While the same trend is observed for all probes used the quantitative shift magnitudes for some shifts differ more than the estimated uncertainties of the fit.

To evaluate the influence of the different scattering powers of the probes on the shift magnitudes, we use our simplistic model: From the simulated model the diffuse x-ray, neutron and electron scattering can be calculated in the kinematical approximation from the same model crystal, eliminating the effect of variances in the sample material. Furthermore, the Bragg scattering in the simulation is limited to one voxel per Bragg-peak, eliminating effects of experimental broadening that could influence the punch and fill routine. The resulting shift magnitudes Δ^+_{OO}, Δ^-_{OO}, Δ^-_{OM} and Δ^+_{MM} from the 3D-ΔPDFs calculated from the model are shown in Supplementary Figure 17. Δ^+_{OM} was omitted in this display as the refined shift magnitude was 0 within the fit uncertainty for all three probes. For Δ^+_{MM} only the analysis based on x-ray and electron diffraction is shown, as for these probes we expect the metal ions to dominate the diffraction experiment, while for neutron diffraction we expect a significant contribution of the oxygen-vacancy correlations.

For Δ^-_{OO}, Δ^-_{OM} and Δ^+_{MM} the observed variances in the shift magnitudes from the calculated 3D-ΔPDFs are on the same order of magnitude as observed experimentally - where the electron and x-ray shift are within the uncertainty of each other while the neutron shift shows more significant deviations. This underlines our argument in the main text that the primary cause for the deviations is related to the different scattering powers.

The analysis of Δ^+_{OO} shows even more drastic differences. Here the influence of the different scattering powers is most severe: Compared to correlations that involve metals, the oxygen-vacancy correlations are much weaker for X-ray and electron diffraction experiments and hence will be more severely influenced by noise. In the simulations, noise is not considered and has no influence, which explains the larger deviations observed in for Δ^+_{OO} in Supplementary Figure 17.

Supplementary Figure 17: Shift positions determined from the model 3D-ΔPDF. Shifts of the observed maxima (red) and minima (blue) away from the average interatomic distance derived from the 3D-ΔPDFs of the calculated models. Shifts are estimated by fitting a three-dimensional Gaussian distribution to the model data displayed in Supplementary Figure 14. Positive shift magnitudes correspond to shifts away from the centre of real space, i.e., elongated interatomic distances. Error bars indicate the 3σ level of the fit uncertainty.

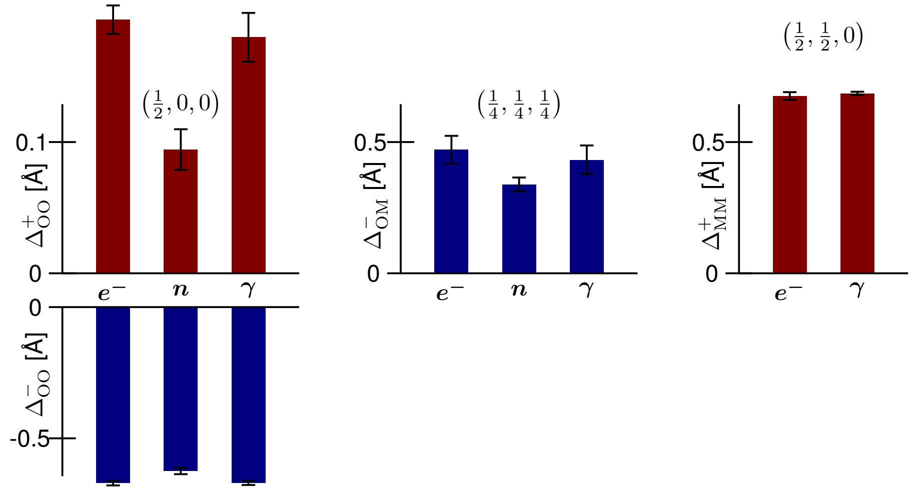

Determination of shift magnitudes from maxima and minima in the 3D-ΔPDF

The nature of the local order in YSZ with predominant static displacements of oxygen ions neighbouring vacancies along $\langle 100\rangle$ towards the vacancy and metal ions neighbouring vacancies along $\langle 111\rangle$ away from the vacancy enable the direct shift magnitude determination from the densities observed in the 3D-ΔPDF as presented in the main text. To demonstrate this fact we consider a simplistic one-dimensional disorder model with 500 000 unit cells and one oxygen atom per unit cell. 5 % of the oxygen atoms are then replaced by vacancies and for every vacancy that was introduced in the structure two oxygen atoms were displaced by $\pm\Delta$. To mimic the average interatomic scattering density these displacements were introduced at random, while in the example for correlated local order as is the case for YSZ the oxygen ions neighbouring the vacancy were shifted towards this vacancy. To mimic the influence of atomic vibrations additionally all atoms were shifted from their position by a Gaussian distributed random number. The corresponding distribution of the first-neighbour scattering density for the average structure and correlated local order case are shown in Supplementary Figure 18(a) for two shift magnitudes

$\Delta_1 = 0.05$ r.l.u. and $\Delta_2 = 0.1$ r.l.u.

The corresponding ΔPDFs for the correlated local order case can be estimated by subtracting the average structure scattering density from the local order scattering density. The resulting ΔPDFs are shown in Supplementary Figure 18(b). Due to the special case of local order present here the position of the minimum directly corresponds to the shift magnitudes and demonstrates why in the case of YSZ the shift magnitude Δ^-_{OO} can be directly derived from the 3D-ΔPDF.

Supplementary Figure 18: Determination of the position of the minima in the 3D-ΔPDF from a one-dimensional simplistic model. (a) Scattering density of the average structure (solid line) compared to short range ordered structure (dashed line) with static displacements as described in the text. Blue: shift magnitude $\Delta = 0.05$ r.l.u., Red: shift magnitude $\Delta = 0.10$ r.l.u. (b) Corresponding ΔPDFs where the position of the minimum is directly related to the shift magnitude Δ.

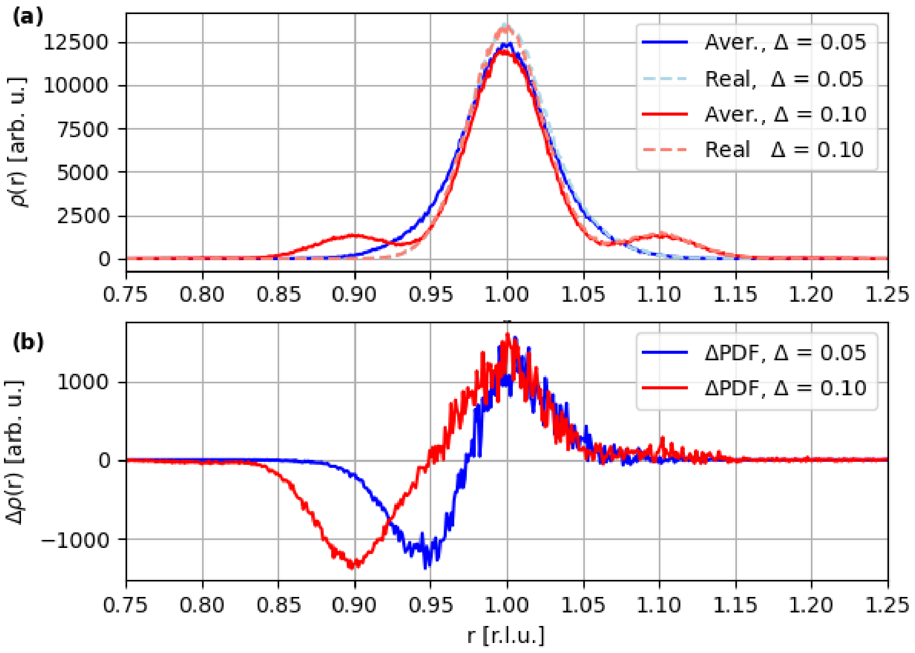

A similar argument holds for the derivation of the metal shift magnitudes derived from the $\langle\frac{1}{4}\frac{1}{4}\frac{1}{4}\rangle$ and $\langle\frac{1}{2}\frac{1}{2}0\rangle$ interatomic vectors. It should be noted however that this procedure in general is only applicable to disorder cases with comparatively large static displacements along certain

directions, as demonstrated here. In the more general case a more sophisticated modelling approach is needed, e.g. using the program YELL[S4] to refine pair correlations or by the explicit realization of a local order model using a direct Monte Carlo simulation[S1].

Supplementary References

[1] Weng, J. *et al.* K-space algorithmic reconstruction (karen): a robust statistical methodology to separate Bragg and diffuse scattering. *Journal of Applied Crystallography* **53**(1), 159–169 (2020).

[2] Virtanen, Pauli, *et al.* SciPy 1.0: fundamental algorithms for scientific computing in Python. *Nature methods* **17**(3)3, 261-272 (2020

[3] Schmidt, E. M. *et al.* three-dimensional difference pair distribution functions (3D-ΔPDFs) of yttria-stabilized zirconia. *Acta Crystallographic Section B.* **79**(2), 138-147 (2023) .

[4] Neder, R. B. & Proffen, T. *Diffuse Scattering and Defect Structure Simulations*, vol. 11. Oxford University Press. (2008).

[5] Prince, Edward, ed. *International Tables for Crystallography, Volume C: Mathematical, physical and chemical tables.* Springer Science & Business Media. (2004).

[6] Simonov, A. *et al.* Yell: a computer program for diffuse scattering analysis via three-dimensional delta pair distribution function refinement. *Journal of Applied Crystallography* **47**(3), 1146–1152 (2014).).
